# Learning from patient safety incidents: The Green Cross method

**DOI:** 10.1111/nicc.13114

**Published:** 2024-06-26

**Authors:** Hilde Kristin Jacobsen, Randi Ballangrud, Gørill Helen Birkeli

**Affiliations:** ^1^ Neonatal Intensive Care Unit Akershus University Hospital Nordbyhagen Norway; ^2^ Department of Health Science Gjøvik Norwegian University of Science and Technology Gjøvik Norway; ^3^ Postanesthesia Care Unit Akershus University Hospital Nordbyhagen Norway; ^4^ Present address: Department of Behavioral Medicine, Faculty of Medicine University of Oslo Oslo Norway; ^5^ Present address: Division of Surgery Akershus University Hospital Nordbyhagen Norway

**Keywords:** critical care nursing, learning health system, patient safety, quality improvement, risk management

## Abstract

**Background:**

Hospitals can improve how they learn from patient safety incidents. The Green Cross method, a proactive reporting and learning method, is one strategy to meet this challenge. In it, nurses play a key role. However, describing its impact on learning from the users' perspective is important.

**Aim:**

This study aimed to describe nurses' experiences of learning from patient safety incidents before and 3 months after implementing the Green Cross method in a postanaesthesia care unit.

**Study Design:**

A qualitative study with an inductive descriptive design with focus group interviews was conducted before and 3 months after implementing the Green Cross method to assess its impact. The data were analysed using qualitative content analysis. The study was conducted in a postanaesthesia care unit in a Norwegian hospital trust.

**Results:**

Before implementing the Green Cross method, participants indicated limited openness and learning, including the subcategories ‘Lack of openness hampers learning’, ‘Adverse events were taken seriously’ and ‘Insufficient visible improvements’. After implementing the Green Cross method, participants indicated the emergence of a learning environment, including the subcategories ‘Transparency increases learning’, ‘Increased patient safety awareness’ and ‘Committed to quality improvements’.

**Conclusions:**

Implementing the Green Cross method in a postanaesthesia care unit positively impacted openness and nurses' patient safety awareness, which is crucial for learning and improving quality.

**Relevance to Clinical Practice:**

The Green Cross method could be useful for organizational learning and facilitating learning from patient safety incidents through transparency, discussion and involvement of nursing staff.

**What is known about the topic**

- Learning from patient safety incidents is challenging and difficult to achieve and is rarely facilitated by reporting systems alone.
- Nurses are rarely involved in the learning process following patient safety incidents.

**What this paper adds**

- Nurses found that the Green Cross method facilitated transparency and patient safety discussions, raising daily awareness of patient safety among nursing staff.
- The Green Cross method was useful in facilitating learning from patient safety incidents by involving nurses in the learning process daily.
- Visible improvements were not fully achieved using the Green Cross method.

## 1 INTRODUCTION

Identifying and learning from factors that contribute to patient safety incidents (PSIs), ‘an event or circumstance which could have resulted or did result in unnecessary harm to patients’,^1^ are essential for developing viable solutions for safer health care.^2^, ^3^ Approximately, 3%–25% of hospitalized patients experience a harmful incident (adverse event [AE]), and 34%–83% of these are considered preventable.^4^, ^5^, ^6^ AEs are a burden to patients, their families and health care professionals and represent a huge health care cost worldwide, becoming a barrier to achieving universal health coverage.^7^, ^8^, ^9^, ^10^

The World Health Organization (WHO) has emphasized the importance of learning from PSIs.^8^, ^11^ Although patient safety learning systems aim to facilitate actionable learning from PSIs, this has proven difficult.^11^, ^12^ Learning from PSIs was also identified as an area for potential improvement in Norwegian intensive care units and postanaesthesia care units (PACUs).^13^ Critical care nurses and registered nurses, hereinafter referred to as nurses, play an essential role in eliminating AEs and keeping patients safe.^8^, ^14^, ^15^ Nurses in the PACU care for patients during the immediate postoperative period,^16^ which entails a significant risk of critical complications.^16^, ^17^ Research shows that AEs most frequently occur in the surgical setting.^4^, ^5^ Therefore, optimizing learning from PSIs in the PACU is critical.

Moreover, health care professionals need a structured method to report and discuss safety issues.^18^, ^19^ In 2011, Södra Älvsborg Hospital in Sweden developed the Green Cross (GC) method,^20^ which is now used internationally.^21^ The GC method is a proactive and user‐friendly incident reporting and learning method that enables health care professionals to identify PSIs daily. Establishing daily GC safety huddles and weekly interprofessional meetings supports collaborative learning by allowing health care professionals to discuss PSI severity and areas for improvement focus.^20^, ^22^ A monthly summary clarifies the results and identifies areas for further improvement work. Specifically, each day of the month is displayed in a cross shape and evaluated with a colour‐coded system indicating the severity level of PSIs. Green indicates that no PSIs occurred, whereas red indicates a serious, avoidable patient injury. Identified risks and AEs initiate daily systematic improvement work. Monthly summaries form the basis for long‐term measures. For a more detailed description of the GC working process, see Birkeli et al.^23^ Unlike traditional incident reporting systems, PSIs are addressed in close relation to where and when they occur and are teamwork based. This requires a safe environment to speak up and a system focus, not unlike the Just Culture.^24^, ^25^, ^26^

### 1.1 Aims and objectives

A PACU in a Norwegian university hospital wanted to improve reporting and learning from PSIs by implementing a modified version of the GC method. This quality improvement (QI) project is the basis for our study, and this is the second paper from the QI project. The first study examined nurses' experience of incident reporting.^23^ This study aimed to describe nurses' experiences of learning from PSIs before and 3 months after implementing the GC method in a PACU.

## 2 DESIGN AND METHODS

To describe nurses' experiences, a qualitative study with an inductive descriptive design with focus group interviews was conducted.^27^, ^28^ All focus group interviews were audiotaped and transcribed verbatim. The data were analysed using qualitative content analysis, according to Graneheim and Lundman.^29^ This paper followed the SQUIRE^30^ and COREQ^31^ guidelines.

### 2.1 Context

The GC method was implemented in a PACU at a university hospital in South‐Eastern Norway. The unit had 25 beds, admitting approximately 1000 patients per month, operating 24/7. Most patients were postoperative, aged 1 year and older. Other patients included adult trauma patients and intermediate or intensive care patients, some requiring overnight care. Each year, 149 patients required non‐invasive ventilation and active cardiovascular physiology management, and 88 were mechanically ventilated. Health care professionals working in the PACU included 40 critical care nurses (post‐graduate registered nurse/Master of Science in Nursing) and 40 registered nurses (Bachelor of Science in Nursing), (with a gender ratio of 90% women and 10% men in total), two nurse managers, three clinical nurse specialists (responsible for training, consultancy and research), three certified nursing assistants, a senior anaesthetist and a pharmacist.

### 2.2 Participants

Nurses were recruited for the focus group interviews using purposive sampling.^28^ Inclusion criteria were frontline nurses working at least 50% part‐time in the PACU for 1 year or more. Staff meetings, emails and verbal reminders during shifts provided information about the study and an invitation to join the focus groups. Sixty‐one nurses met the inclusion criteria and were invited to participate. In all, 22 nurses participated in focus group interviews before and after implementing the GC method, with 13 nurses involved both before and after the implementation (Table 1). Participants in focus group interviews before implementation were younger and had less clinical experience than those after implementation. Reasons for not participating in focus groups included a busy PACU, time off, illness and a new job.

**TABLE 1:** Participant characteristics in focus groups before and after implementation of the Green Cross method (*n* = 22).

Characteristic	Before implementation		Three months after implementation		Both before and 3 months after implementation		Total	
	*n* = 19	%	*n* = 16	%	*n* = 13	%	*n* = 22	%
Sex								
Female	14	74	15	94	12	92	17	77
Male	5	26	1	6	1	8	5	23
Age (years)								
23–39	9	47	4	25	4	31	9	41
40–49	7	37	7	44	6	46	8	36
50–69	3	16	5	31	3	23	5	23
Title								
RN	8	42	4	25	4	31	8	36
CCN	11	58	12	75	9	69	14	64
Years of clinical experience								
1–10 years	7	37	3	19	3	23	7	32
11–25 years	9	47	9	56	7	54	11	50
26 years or more	3	16	4	25	3	23	4	18
Years of employment in the PACU								
1–5 years	13	68	9	56	8	62	14	64
6–25 years	4	21	4	25	3	23	5	23
26 years or more	2	11	3	19	2	15	3	14
Working position								
Part‐time	3	16	3	19	2	15	4	18
Full time	16	84	13	81	11	85	18	82

Abbreviations: CCN, critical care nurse; PACU, postanaesthesia care unit; RN, registered nurse.

### 2.3 Planning and implementation

To address the shortcomings in reporting and learning from PSIs, the PACU management considered the GC method the most appropriate tool. Planning and implementing the GC method working process were assigned to a clinical nurse specialist (last author). The GC method was modified to fit the PACU setting (Table 2).^20^, ^32^ The nurses who identified a PSI documented it anonymously or signed in a detailed report form.

**TABLE 2:** The modified version of the Green Cross method working process, as implemented in this study.

1. Identification	PSIs are documented by the health care professional who discovered the incident using the detailed report form. Documentation can be anonymous or signed. All events documented in the previous 24 h are read aloud each morning to the nurses, and each afternoon to the anaesthetists.
2. Assessment of seriousness	The reporting health care professional assesses the event's severity before the health care team discusses it. Once the severity has been agreed upon, it is illustrated on the basic green cross template using the colour code below. Reproduced with permission from Källman et al.^20^ 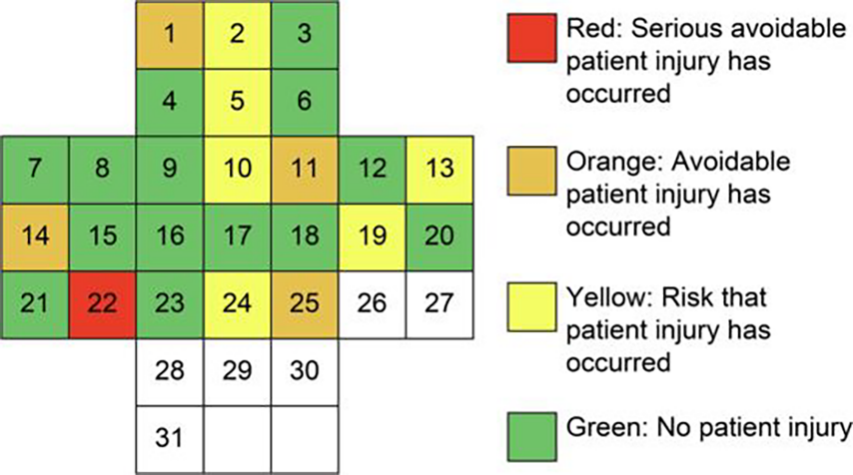
3. Non‐conformance reporting	For serious events (red/orange), the health care professional must file a report to the hospital's electronic incident report system.
4. Improvement of work and interprofessional weekly meetings	Daily improvement work is systematic. Risks are dealt with as soon as they are identified or in the 30‐min weekly interprofessional meetings.
5. Follow‐up and learning	Follow‐up takes place during daily improvement work and weekly interprofessional improvement meetings. All events described in detailed report forms are summarized monthly to identify problem areas and visualize the outcome. Based on these summaries, long‐term actions are taken to prevent recurrence. The GC template and the monthly summary raise awareness of focus areas in the PACU to improve patient safety.

Abbreviations: GC, Green Cross; PACU, postanaesthesia care unit; PSI, patient safety incident.

Kotter's 8‐Step Process for Leading Change was used to create a climate of change and ensure a common understanding, staff involvement and change sustainability.^32^ This process emphasizes the importance of tailoring improvements; therefore, the implementation of the CG method in this study differed slightly from the original Swedish method.^20^ The eight steps were as follows; (1) *create a sense of urgency*. Both nurses and management wanted to increase reporting and learning from PSIs. (2) *Build a guiding coalition*. A project group was formed and met weekly. (3) *Form a strategic vision*. Zero‐vision for PSIs agreed upon, project plan created and GC method^33^ adapted to the PACU. (4) *Enlist a volunteer army*. Nurses received a 2‐h lecture on the GC method and QI work, and information through email and staff meetings. (5) *Enable action by removing barriers*. Reports were visible, daily and weekly meetings were introduced and a GC summary was sent in weekly emails. (6) *Generate short‐term wins*. GC kick‐off and milestones were celebrated, and QIs were made visible. (7) *Sustain acceleration*. The PACU management was present daily, emphasizing the importance of the GC method. The project group repeatedly asked if anyone had anything to report and met weekly for status and problem‐solving. (8) *Institute change*. Implementation continues. Daily, monthly and annual follow‐ups are necessary for the method to become culturally embedded.

### 2.4 Data collection

Exploratory focus group interviews based on the same semi‐structured thematic interview guide were conducted before and 3 months after implementing the GC method.^27^, ^28^ The interview guide included seven open‐ended qualitative questions^27^ (Supplementary file 1) and was validated based on a pilot focus group interview, after which one question was revised.^28^ The same interview guide was used for all focus group interviews. Questions related to learning were ‘When patient safety incidents occur in your unit, how do you learn from them?’ ‘When patient safety incidents are reported, how are they handled?’ ‘How are medical errors handled in your unit?’ ‘How do you assess patient safety in your unit?’ and ‘What is your experience of the Green Cross method?’. Follow‐up questions such as ‘Could you please explain more …?’ and ‘When you say…what do you mean?’ were used to elaborate and clarify the answers. Four focus group interviews were conducted in January 2019, and four follow‐up focus group interviews were conducted in May 2019. All interviews occurred during shifts in a room near the PACU^34^, ^35^ with three to six participants.^28^, ^34^ Focus group interviews lasted from 43 min to 1 h 39 min (mean of 1 h 8 min). Each interview was moderated by the first author, a trained facilitator working as a clinical nurse specialist in the neonatal intensive care unit with no connection to the PACU. The last author, a clinical nurse specialist in the PACU, took observational notes.^28^ All focus group interviews were audiotaped, transcribed verbatim by the first and last authors and de‐identified before analysis.

### 2.5 Data analysis

Following the data collection, a qualitative manifest and inductive content analysis was conducted as described by Graneheim and Lundman.^29^, ^36^ A manifest inductive approach is text‐driven and characterized by a search for patterns. The researcher seeks similarities and differences during the analysis, creating categories at various levels of abstraction.^36^ Thus, the categories and the subcategories represent the manifest text content.^29^ All transcriptions were read multiple times to ensure accuracy and an overview of the data. The data were then structured into meaning units (*n* = 620), condensed and abbreviated without losing essence. Codes were constructed from the meaning units, and codes with identical or equivalent characteristics were grouped into subcategories using Microsoft Excel.^37^ The first and last authors created subcategories individually before reaching a consensus through discussion. The subcategories were grouped into four mutually exclusive categories, two related to the learning aspect. The data were analysed using a back‐and‐forth process. To increase the credibility of the findings, discussions were held among all three authors throughout the data analysis.^38^

## 3 ETHICAL CONSIDERATIONS

This QI project was approved by the data protection officer (ref. 69_2018) and the PACU management. Because of the nature and design of this study, approval from The Norwegian Centre for Research Data was not required (ref. 606302). The QI project was conducted following the Ethical guidelines for nursing research in the Nordic countries^39^ and the Declaration of Helsinki.^40^ The ethical principles of autonomy, beneficence, justice and nonmaleficence were ensured through voluntariness, confidentiality, written informed consent, the right to withdraw without reason or consequence and an invitation to all who met the inclusion criteria to participate. An external counsellor was available if needed.^39^, ^41^

## 4 FINDINGS

The data analysis identified two categories. Before implementing the GC method, the category ‘Limited openness and learning’ was constructed, consisting of three subcategories (Table 3). Three months after implementation, the category indicating ‘A learning environment emerging’ was constructed, consisting of three subcategories (Table 4).

**TABLE 3:** Findings before implementing the Green Cross method.

Subcategory	Category
Lack of openness hampers learning	Limited openness and learning
Adverse events were taken seriously	
Insufficient visible improvements	

**TABLE 4:** Findings 3 months after implementing the Green Cross method.

Subcategory	Category
Transparency increases learning	A learning environment is emerging
Increased patient safety awareness	
Committed to quality improvements	

### 4.1 Limited openness and learning (before implementing the GC method)

This category described nurses' experience in a PACU environment where communication and learning from PSIs were insufficient. This category comprised three subcategories (see Table 3).

#### 4.1.1 Lack of openness hampers learning

Nurses had limited knowledge of what PSIs had been reported and what actions had been taken to address them. ‘I can't remember ever coming to work where PSIs were discussed and suggestions for improvement were made’ (5). They experienced the feedback in the hospital's mandatory reporting system as a closed loop between the management and the reporter, hampering learning from PSIs.

They (the management) don't talk much about […] what PSIs are reported. And do they act on them? It goes from you to the management and back to you… then you're the only one who knows anything about it' (3).

Occasionally, the most serious incidents were shared by email. However, nurses expressed that they needed greater openness about all reported PSIs to enable learning for everyone. ‘I think there are things (PSIs) that […] need to be shared so we can learn more (from them)’ (4).

#### 4.1.2 AEs were taken seriously

Nurses experienced limited openness about PSIs. However, the management shared information about reported AEs and actions taken through staff meetings, emails, morning meetings and posters. When asked how medical errors are dealt with in the unit, one nurse replied:

If there are serious incidents involving equipment or medication, that's probably where most of the feedback (from the management) is […] otherwise, it feels like they (PSIs) are just being put in a drawer (17).

Nurses expressed that feedback from the management was positive and raised awareness. However, organizational learning was limited.

#### 4.1.3 Insufficient visible improvements

Nurses described a situation with insufficient visible improvements or solutions to the reported PSIs. Minor incidents and PSIs involving other units showed little or no improvement. When asked how they learn from PSIs, one nurse responded: ‘We've reported a lot, but it doesn't get any better […], after surgery all patients still come without (intravenous cannula) caps’ (18). This was a common theme in all the focus group interviews. Nurses were often unaware when action was taken. They experienced a lack of common understanding and standards and wished for better communication between units.

### 4.2 A learning environment emerging (3 months after implementing the GC method)

This category described a PACU where nurses experienced increased transparency regarding PSIs after implementing the GC method. Both management and nurses focused more on improvement, enabling learning from PSIs. However, the complex hospital organization made it difficult to follow through. This category comprised three subcategories (see Table 4).

#### 4.2.1 Transparency increases learning

After implementing the GC method, nurses experienced increased transparency and received more information about PSIs. They found the daily review of reported incidents useful reminders that increased their awareness of incidents and ability to learn from them.

I think when yesterday's incidents are read […], it puts focus on them […] and you are more alert that day (20).

In addition, the management provided weekly emails with a summary of PSIs, interprofessional discussions and ongoing improvements. Nurses stated that feedback was continuous, with improvements communicated daily and weekly. When asked, ‘What is your experience of the Green Cross method?’ one nurse responded:

I'm very positive about the Green Cross, and I spend time reading the weekly emails […] even though I'm not at work, I learn from it…everyone learns from it (25).

#### 4.2.2 Increased patient safety awareness

Implementing the GC method facilitated weekly interprofessional meetings to discuss PSIs and work on improvements. Nurses felt that the reported PSIs were taken seriously and that it was easier to talk about PSIs because it highlighted the factors leading to incidents rather than the people involved. Learning from each other rather than exposing each other was the main goal.

Fortunately, medication errors don't happen very often […], so it's nice to hear about someone else's mistakes… You don't have to make a mistake yourself […], but (you) realize… I must be more aware now (24).

When asked how they assessed patient safety in the unit, one nurse elaborated:

With the Green Cross, it (patient safety) is even better […], a greater focus on quality and how to do the best for our patients […], and more transparency […]. It shows that we are working every day to improve (35).

Overall, nurses expressed an increased awareness of PSIs, their role in patient safety and how they can contribute to patient safety in general.

#### 4.2.3 Committed to QI

Nurses experienced increased focus and commitment to QI among nurses, management and other units after implementing the GC method.

I think […] that you should be proud of the workplace using the Green Cross… It's visible to everyone that we're working systematically to improve (35).

However, the nurses found it difficult and time‐consuming to influence the complex hospital system and improvements in other units. Nevertheless, they expressed that reporting using both the GC and the hospital's reporting system increased the possibility of improvement and improved learning from PSIs. As a nurse (26). Communicated, ‘Staffing is a very difficult issue, and it probably takes a very long time to fix; the Green Cross may help’.

## 5 DISCUSSION

This study aimed to describe nurses' experiences of learning from PSIs before and 3 months after implementing the GC method in a PACU. Before implementation, nurses experienced limited openness and learning about PSIs. Although AEs were taken seriously, more transparency and visible improvements were desired. After implementation, the nurses reported that a learning environment was emerging. Increased transparency and discussion about PSIs and increased awareness of patient safety in the PACU facilitated learning. However, the nurses still demanded more visible improvements.

### 5.1 Limited openness and learning

Nurses in the present study experienced a lack of openness that hampered learning before implementing the GC method. They had limited knowledge about PSIs reported in the PACU and described the current reporting system as a closed loop between the reporter and management, thus hindering others' ability to learn. This aligns with a British hospital‐based study indicating that reporting systems do not facilitate learning or shared learning.^42^ Nurses in the present study expressed frustration because of lack of feedback and inadequate involvement in the learning process after reporting the incidents. The British study found that nurses experience insufficient feedback.^42^ Moreover, a Danish study reported that frontline staff rarely participated in the learning process after PSIs.^43^ Nurses in the present study called for openness about all reported PSIs and actions taken. Despite management sharing summarized information about the AEs and addressing them accordingly, the nurses did not feel that this enhanced organizational learning. They desired common arenas where all health care professionals can share and discuss PSIs to facilitate learning. Sharing PSIs is crucial to achieving organizational learning.^44^ According to Peter Senge,^45^ decentralizing leadership can enhance people's capacity to collaborate towards common goals. This is an important step towards creating a learning organization. Hospitals can achieve comprehensive and sustainable improvements in quality and safety^7^, ^15^ by providing learning arenas for interprofessional teams to discuss PSIs and patient safety.^8^, ^19^

Insufficient visible improvements were a source of frustration among nurses before implementing the GC method. They reported frequently, but it did not improve. This is consistent with previous Norwegian studies^13^, ^46^ and the WHO reports.^11^ It is often called the ‘knowing‐doing gap’ because of slow uptake.^8^ The lack of visible improvements is well documented^47^ and contributes to stagnating progress in patient safety.^48^, ^49^, ^50^ Reported incidents must be linked to visible actions to prevent recurrent PSIs and improve patient safety.^47^, ^51^, ^52^

### 5.2 A learning environment emerging

After implementing the GC method, nurses described that transparency increases learning. The daily safety huddles and weekly interprofessional meetings provided information about which PSIs were reported and they could discuss how to prevent the same PSIs from reoccurring. A British study^25^ shows that the quality of feedback given to those who report the PSIs is critical to enable learning. This is further supported by a Delphi consensus process involving 15 international patient safety experts.^53^ In the present study, weekly emails summarizing the previous week's PSIs and improvement work enabled all nurses, even those absent from work, to be aware of and learn from reported incidents. Email is often used to provide feedback to frontline staff, communicate actions taken and raise risk awareness.^52^, ^54^ However emails and creating new guidelines have proven to be relatively weak change strategies.^11^

Nonetheless, nurses found that the daily safety huddles and weekly interprofessional meetings provided by the GC method for sharing and discussing PSIs increased patient safety awareness. This is supported by two Swedish studies^20^, ^22^ and is consistent with previous findings in a British study^25^ that discussing PSIs was perceived to positively affect patient safety through involvement and knowledge. This is an essential part of the GC method.^20^ However, the interprofessional meetings were more difficult to attend than the daily safety huddles because of the hectic and unpredictable nature of the PACU. Safety huddles are recommended by The Joint Commission and can significantly improve patient outcomes and the patient safety culture.^55^, ^56^ Hearing about others' mistakes made nurses in present study more aware of PSIs. This finding is consistent with a previous Swedish study.^22^ They became more alert and adjusted their work to control the situation and prevent the same PSI from recurring. Modifying actions to avoid errors, new work routines and procedures are common but ineffective ways to deal with errors.^57^, ^58^, ^59^ Most of the interventions identified in the present study were such adaptations. They may partly explain why nurses continued to experience few visible improvements after implementing the GC method.

Nurses felt more committed to QI after implementing the GC method. The GC method symbolized that they took patient safety seriously in their unit, were proud of it and desired further improvement. Nurses emphasized the empowering effect of the GC method, which enabled the interprofessional team to collaborate to identify areas for improvement, providing a basis for improvement work.^32^ These findings suggest increased organizational learning after implementing the GC method. They are supported by a previous Swedish study, which found that organizational learning and continuous improvement were rated significantly higher in GC units than in non‐GC units.^20^ In the present study, nurses reported difficulties in making improvements involving other units and found the system too complex to change. This is in line with studies conducted in Australia,^60^ Sweden^61^ and The United Kingdom.^62^ In clinical practice, nurses must engage in QI. However, nurses and other health care professionals have been poorly educated on undertaking QI.^63^, ^64^, ^65^ To do successful QI, skills in conducting improvement work are needed.^65^

Identifying efficient methods to change practices was challenging in the present study. This aligns with worldwide challenges in improving patient safety through learning from PSIs.^8^, ^11^ Redesigning a health care system that enables everyone to participate in daily improvements, thus becoming a learning organization, is challenging.^45^ A learning organization commits to systems thinking, personal mastery, mental models, building shared vision and team learning.^45^ All these dimensions were facilitated by the GC method. Nurses in the present study were motivated by visible improvements but still found them insufficient. They perceived visible improvements were more likely if PSIs were reported in both the hospital's incident reporting system and the GC. However, a positive focus on improving patient safety through learning from successes, in addition to learning from failure, has been suggested.^8^, ^66^, ^67^

### 5.3 Limitations

This study had some limitations. A modified version of the GC method was used; accordingly, the findings may not be transferable to other units. GB, who observed the focus groups, worked in the PACU and as the QI project manager. This may have prevented participants from speaking freely and influenced the findings. Additionally, only nurses were included in the focus groups. Including other health care professionals may have yielded different findings. Furthermore, the participants were not the same before and after the implementation. Only a few nurses participated in both focus groups, which may have influenced the findings and the trustworthiness of the study.^38^ Data collection and analysis occurred 4 years ago; however, the findings might provide valuable in‐depth knowledge about nurses' experience of learning from PSIs using the GC method.

### 5.4 Implications for practice

The findings of this study may have significance for leaders, offering insights into improving learning from PSIs by actively engaging frontline staff. The GC method facilitated interprofessional collaboration to learn from PSIs, thus improving patient safety. Nurses experienced increased patient safety awareness and an enhanced engagement in QI work when using the GC method. This represents a positive move towards becoming a learning organization. However, future studies should investigate the potential benefits of combining the GC method with learning from excellence.

## 6 CONCLUSIONS

Implementing the GC method resulted in daily disclosure of near misses and errors. This increased the nurse's patient safety awareness and facilitated learning from each other's experiences. Although visible improvements remained insufficient, the GC method increased nurses' commitment to QI. These findings indicate that the GC method positively impacts openness and safety awareness, which are vital for learning and QIs.

## AUTHOR CONTRIBUTIONS

Hilde Kristin Jacobsen, Gørill Helen Birkeli and Randi Ballangrud were responsible for the study design. Hilde Kristin Jacobsen and Gørill Helen Birkeli performed the data collection and data analysis. Randi Ballangrud contributed to the data analysis. All authors contributed to drafting of the manuscript, revising it critically for important intellectual content and reading and approving the final manuscript.

## Supporting information


**Data S1.** Supporting information.

## Data Availability

The data that support the findings of this study are available on request from the corresponding author. The data are not publicly available due to privacy or ethical restrictions.
