# Supplementary material for: Learning from patient safety incidents: The Green Cross method
Source: Nurs Crit Care. 2024 Jun 26;30(2):e13114. doi: 10.1111/nicc.13114 (PMC11873358; doi:10.1111/nicc.13114)
Supplement: Supplementary file 1 — Data S1. Supporting information. [file NICC-30-0-s001.docx]

Interview guide for focus groups before and after the implementation of the Green Cross method

1. What is your experience with the culture of reporting patient safety incidents in your unit? When patient safety incidents are reported, how are they handled?
2. What is your experience of the patient safety incident reporting process in your unit?
3. When patient safety incidents occur in your unit, how do you learn from them?
4. How do you feel your colleagues respond when you raise concerns about patient safety in your unit?
5. How are medical errors handled in your unit?
6. How do you assess patient safety in your unit?
7. What is your experience with the Green Cross method?

Follow-up questions related to all questions in the interview guide:

- Could you please tell me more about….?
- When you say…, what do you mean?
- Is it always like this?
- Could you please elaborate?
- Could you please explain more?
